# Supplementary material for: The Effect of Crystallinity on the Toughness of Cast Polyamide 6 Rods with Different Diameters
Source: Polymers (Basel). 2020 Feb 2;12(2):293. doi: 10.3390/polym12020293 (PMC7077395; doi:10.3390/polym12020293)
Supplement: Supplementary file 1 [file polymers-12-00293-s001.zip › dsc curves/pdf/130_I.pdf]

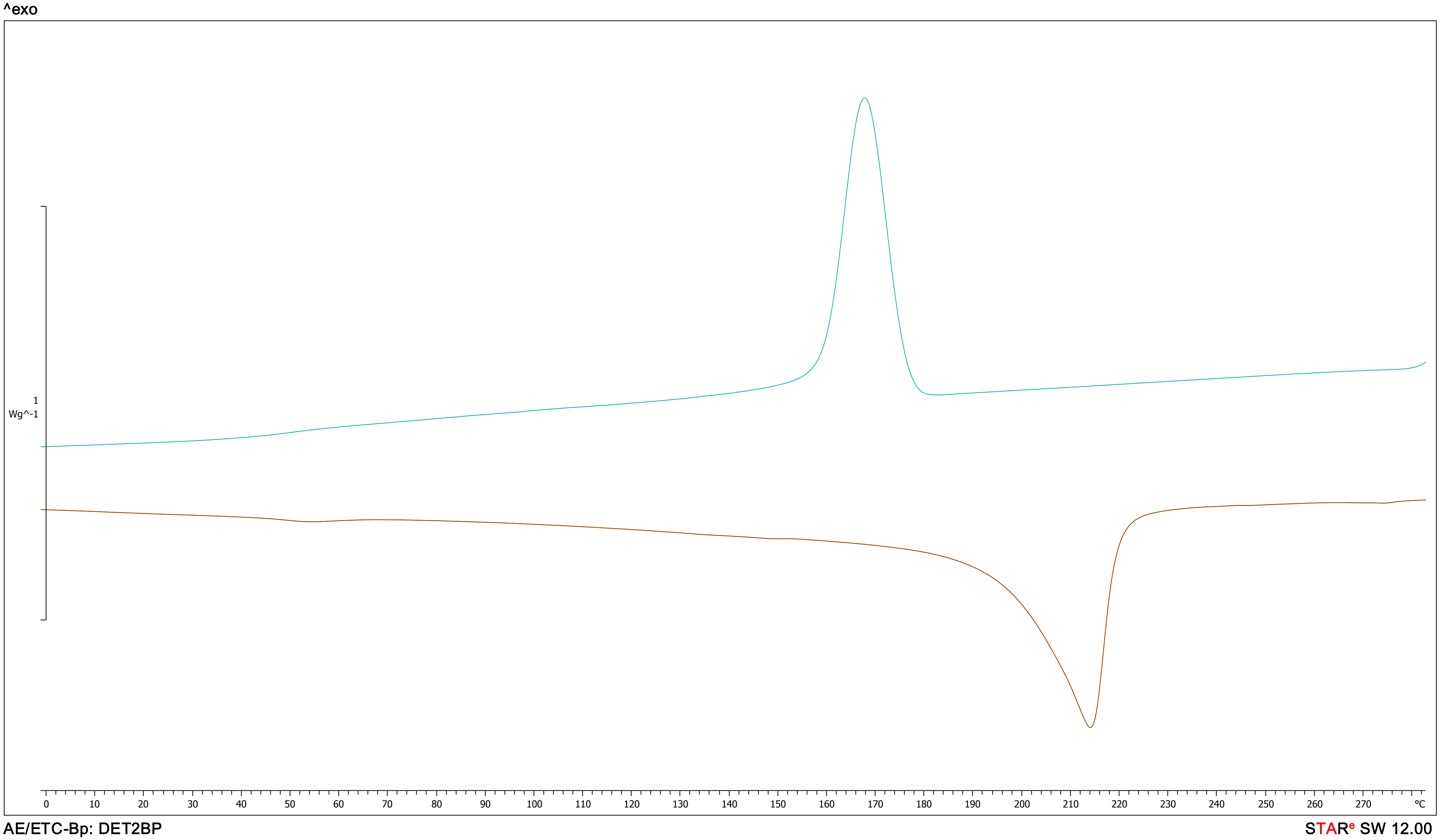

^exo
1
Wg^-1
0 10 20 30 40 50 60 70 80 90 100 110 120 130 140 150 160 170 180 190 200 210 220 230 240 250 260 270 °C
AE/ETC-Bp: DET2BP
STARe SW 12.00
