# Supplementary material for: The Effect of Crystallinity on the Toughness of Cast Polyamide 6 Rods with Different Diameters
Source: Polymers (Basel). 2020 Feb 2;12(2):293. doi: 10.3390/polym12020293 (PMC7077395; doi:10.3390/polym12020293)
Supplement: Supplementary file 1 [file polymers-12-00293-s001.zip › dsc curves/pdf/300_III.pdf]

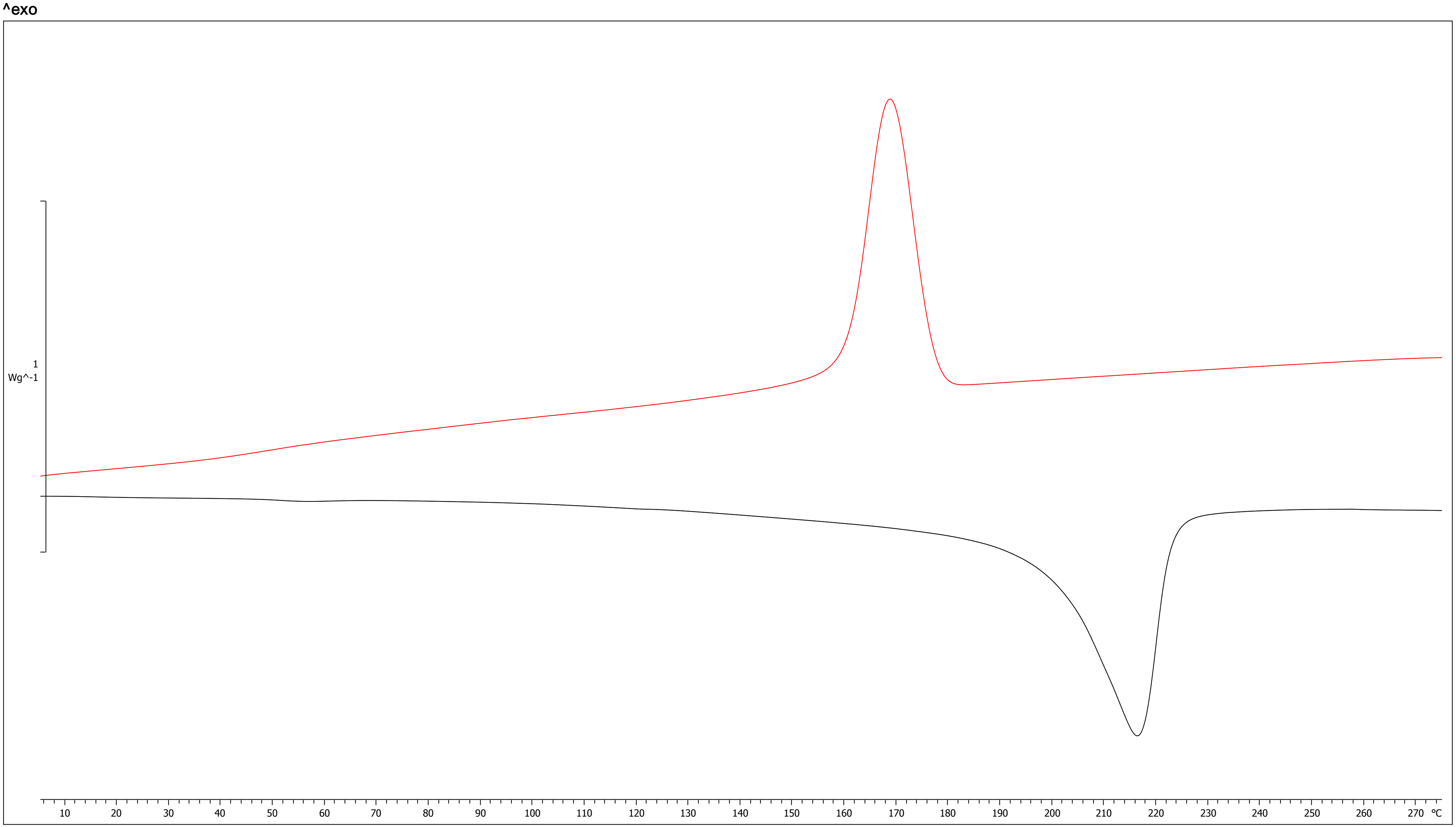

AE/ETC-Bp: DET2BP

STARᵉ SW 12.00
